# ﻿Three new species of the cockroach genus *Nocticola* Bolívar, 1892 (Blattodea, Corydioidea, Nocticolidae) from China

**DOI:** 10.3897/zookeys.1232.136907

**Published:** 2025-03-19

**Authors:** Ting-Ting Li, De-Xing Liu, Jian Chen, Xiao-Ya Wei, Qiao-Yun Yue, De-yi Qiu

**Affiliations:** 1 State Key Laboratory of Medical Vector Surveillance (Guangdong), Technology Center of Zhongshan Customs, Zhongshan, Guangdong 528400, China State Key Laboratory of Medical Vector Surveillance (Guangdong), Technology Center of Zhongshan Customs Zhongshan China; 2 Zhongshan Torch Polytechnic, Zhongshan, Guangdong 528400, China Zhongshan Torch Polytechnic Zhongshan China

**Keywords:** Cave cockroach, epigean, identification key, new species, *Nocticola*, taxonomy

## Abstract

Three new species of *Nocticola* Bolívar, 1982 from Guangxi Province, China are described: *Nocticolabaiguensis***sp. nov.**, *Nocticolacordiformis***sp. nov.**, and *Nocticolaappendiculata***sp. nov.** Morphological features associated with the wings, the specialized abdominal tergum, and genitalia of these new species are described and illustrated in detail. A key to the recorded *Nocticola* species from China is provided in this paper.

## ﻿Introduction

The genus *Nocticola*, with the type species *Nocticolasimoni* Bolívar, was established by Bolívar (1892), who also described *N.caeca* Bolívar, 1892 in the same year; the first family-group name based on *Nocticola* is Nocticolinae (Bolívar 1892). Nocticolidae was first used by Bruner (1915) for two species described by Silvestri (1946), *N.sinensis* Silvestri and *N.termitophila* Silvestri, with *N.sinensis* being the earliest recorded *Nocticola* in China. Currently 29 known *Nocticola* species have been described all over the world (Beccaloni 2014; Trotter et al. 2017; Liu et al. 2017). They are very small and delicate, with reduced male wing veins. Among them, 17 species are cavernicolous, eight epigean, and four termitophilous (Fernando 1957; Roth 1988, 1991, 1995, 2003a; Roth and McGavin 1994; Andersen and Kjaerandsen 1995; Lucañas and Lit 2016; Lucañas et al. 2021; Lucañas and Maosheng 2023; Liu et al. 2017; Trotter et al. 2017). Handlirsch (1925) once classified *Nocticola* as Blattoidea based on the characteristics of the ovipositor valves in the seventh abdominal plate of *Nocticola* females, and subsequently, Princis (1966) listed Nocticolidae as a branch of Blattoidea. Roth (1988) suggested that Nocticolidae could be placed between Polyphagidae and Blattoidea. In recent years, molecular analysis has recovered Nocticolidae as sister to Corydiidae, particularly Latindiinae (Inward et al. 2007, Murienne 2009; Djernæs et al. 2012), supporting the position of Nocticolidae and Latindiinae as sister groups, and infer that Nocticolidae may be a specialized form of Latindiinae (Djernæs et al. 2015; Legendre et al. 2015; Li and Huang 2020). Subsequently, Han et al. (2023) assess the phylogenetic relationships of 35 Corydioidea species with mitochondrial genomes and two nuclear gene fragments, and they infer that the Latindiinae belong to the family Nocticolidae.

## ﻿Materials and methods

### ﻿Morphological study

**Figure 1.**
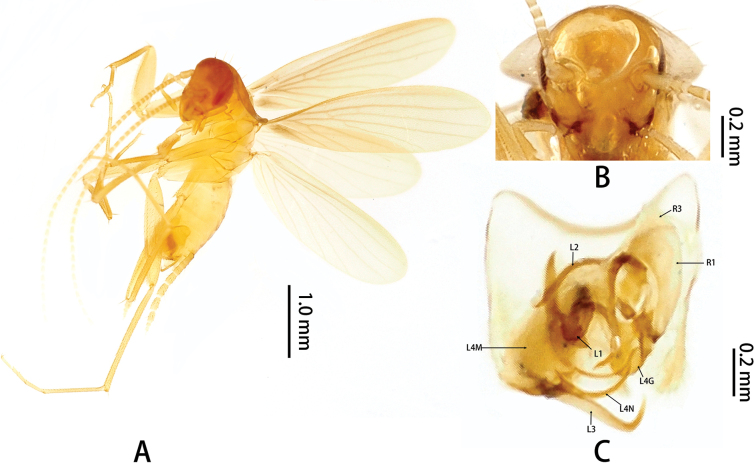
*Nocticolaxiai* Liu, Zhu, Dai & Wang, 2017: **A** holotype male, lateral **B** head **C** phallomeres (photographs provided by Han-Qiang Wang, SHEM). Abbreviations: L1, L2, L3, L4G, L4N, L4M: sclerites of the left phallomere (L1 situated in the central dorsal wall; L2 arch-shaped sclerite situated in the ventral to L1; L3 situated in the left wall protrudes a large hook-process; L4G situated in the posterior ventral wall of the ventral lobe; L4N accessory hook-like phallomere; L4M situated in the ventral wall); R1, R3: sclerites of the left phallomere (R1 situated in the dorsal or ventral walls, or in the posterior part of dorsal and ventral wall; R3 plate-like situated in the anteriormost ventral wall).

**Figure 2.**
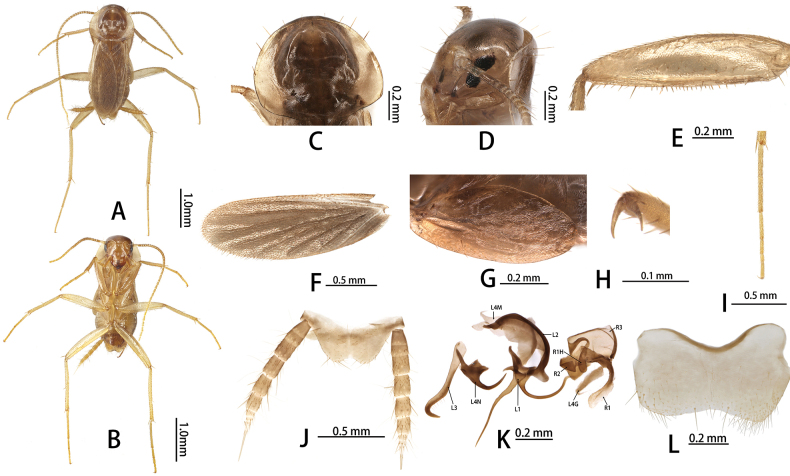
*Nocticolasinensis* Silvestri, 1946: **A** male, dorsal view **B** male, ventral view **C** pronotum **D** head **E** fore femur **F** tegmen **G** hind wings **H** tarsal claw **I** hind tarsus **J** supra-anal plate, dorsal view; male genitalia **K** phallomeres **L** subgenital plate, ventral view. Abbreviations: L1, L2, L3, L4G, L4N, L4M sclerites of the left phallomere (L1 situated in the central dorsal wall; L2 arch-shaped sclerite situated in the ventral to L1; L3 situated in the left wall protrudes a large hook-process; L4G situated in the posterior ventral wall of the ventral lobe; L4N accessory hook-like phallomere; L4M situated in the ventral wall); R1, R2, R3, R1H sclerites of the left phallomere (R1 situated in the dorsal or ventral walls, or in the posterior part of dorsal and ventral wall; R2 a ridge on the ventral margin; R3 plate-like situated in the anteriormost ventral wall; R1H a larger lobed situated in the dorsal wall, with extensions into the ventral wall).

Specimens were collected in Guangxi Province from 2023–2024. The examined material of *N.xiai* Liu, Zhu, Dai & Wang, 2017 (Fig. 1A–C) is deposited in the Institute of Entomology of the Shanghai Entomological Museum, Chinese Academy of Sciences, Shanghai (**SHEM**). The type specimens of the three new species and the examined specimens of *N.sinensis* Silvestri, 1946 (Fig. 2A–L) were deposited in the Zhongshan Customs Technology Center (**ZSCTC**).

The lateral tergum behind the seventh abdominal tergum (T7) of the male specimen was cut off, placed into a 1.5 ml centrifuge tube with 10% NaOH and digested at 70 °C for 30–45 min. After digestion, the NaOH was removed from the centrifuge tube, and the specimen was rinsed thrice with water before examination. The specimens were dissected and observed under a ZEISS Discovery V12 stereo microscope. Photographs were taken with a ZEISS/Smart Zoom5 and Canon EOS 5D Mark III, and illustrated with Adobe Photoshop 2022 software. After illustration, the genitalia were stored in 0.5 ml centrifuge tubes containing 50% glycerol, and preserved with the remainder of the specimen, which was stored in ethyl alcohol. Terminologies used for male genitalia follow Klass (1997) and for other characters follow Roth (2003b).

### ﻿Molecular biology study

DNA was obtained from hind tarsus of adult and nymph cockroaches using TIANamp Genomic DNA Kit produced by Tiangen. The primers used for PCR amplification were the universal primers for cytochrome C oxidase subunit I (COI) gene: LOC1490 5’-GGTCAACAAATCATAAAGATATTGG-3’, HCO2198 5’-TAAACTTCAGGGTGACCAAAAAATCA-3’ (Folmer et al. 1994). The reagents for primers synthesized, EX-Taq DNA polymerase and dNTP were purchased from Takara Biotechnology (Dalian) Co., Ltd. The amplification conditions were denaturation at 95 °C for 5 min; 95 °C for 30 s, 50 °C for 30 s, 72 °C for 60 s, 35 cycles; final extension at 72 °C for 10 min, then held at 4 °C. The amplified samples with bands in Gel imaging System were sequenced by Tianyi Huiyuan Gene Technology Co., Ltd. (Guangzhou).

A total of 20 COI sequences were analyzed, of which 15 sequences were obtained in this study and Five sequences were downloaded from GenBank (Yanagisawa et al. 2021; Han et al. 2023; Li et al. 2022). *Latindia* sp. 1, *Latindia* sp. 2, and *Eucorydiadasytoides* were selected as outgroups (Table 1). Unfortunately, molecular data for *N.xiai* were not obtained, so could not be included in this study. All 15 sequences have been submitted to GenBank (https://www.ncbi.nlm.nih.gov/nuccore) with accession numbers PQ601347–PQ601361. The COI fragments were processed using the MUSCLE algorithm within MEGA 6.0 (Tamura et al. 2013), and a phylogenetic tree was constructed using Maximum Likelihood (ML) tree (Kimura 1980) and visualized using FigTree v. 1.4.4 (http://tree.bio.ed.ac.uk/software/figtree/). Nucleotide sites differences, genetic distances, both interspecific and intraspecific were calculated using the Kimura-2-paramaters (K2P) with 1,000 ultrafast bootstrap replicates (Hoang et al. 2018).

**Table 1.** Samples used in the phylogenetic analysis.

Species	Voucher number	Location	GenBank number
*N.baiguensis* sp. nov.	LI49-1	Yanshan District, Guilin City, Guangxi Province	PQ601347
	LI49-2		PQ601348
	LI69-1	Xiangshan District, Guilin City, Guangxi Province,	PQ601353
	LI69-2		PQ601354
	LI70	Diecai District, Guilin City, Guangxi Province,	PQ601355
	LI71-1		PQ601356
	LI71-2		PQ601357
*N.cordiformis* sp. nov.	LI51	Lingui District, Guilin City, Guangxi Province	PQ601349
	LI85		PQ601352
	LI86		PQ601358
	LI95		PQ601359
	LI96		PQ601360
*N.appendiculata* sp. nov.	LI81	Lingchuan County, Guilin City, Guangxi Province,	PQ601350
	LI82	Lingui District, Guilin City, Guangxi Province	PQ601351
*N.sinensis*	LII16	Jiangyong County, Yongzhou City, Hunan Province	PQ601361
*Nocticola* sp. 3 ZQW-2023c	/	/China	OR478956
*Nocticola* sp. 2 ZQW-2023c	/	/China	OR478955
*Latindia* sp. 1	B467	French Guiana	OL740656
*Latindia* sp. 2	B481	French Guiana	OL810009
*Eucorydiadasytoides*	/	Taiwan, China	LC480880

## ﻿Results

### ﻿Taxonomy

**Family Nocticolidae Bolívar, 1892**

#### ﻿Genus Nocticola

Taxon classificationAnimaliaBlattodeaNocticolidae

Bolívar, 1892

20E84954-832B-51F8-A9F6-F9721D7587E8

Nocticola Bolívar, 1892: 29.

##### Type species.

*Nocticolasimoni*. First used as Nocticolidae Brunner 1915. Roth (1988) diagnosed and discussed Nocticolidae.

##### Diagnosis.

The following description is in accordance with the traits proposed by Roth (1988) and the diagnosis of Andersen and Kjaerandsen (1995). Habitus small and delicate. Eyes well developed, variably reduced or absent; ocelli present or absent. Male wings are either reduced or well developed; front and hind wings are similar if well developed, membranous hyaline with minute pubescence and with few, essentially straight veins. Anteroventral margin of front femur with a row of piliform setae only, terminating at one or more large spines (= Type C); arolia and pulvilli absent; tarsal claws very small, simple, and symmetrical. Left and right phallomeres of the male genitalia are complex, always with a hook. Modification of the male abdomen tergal gland divides *Nocticola* into two groups: the *simoni* species group (male terga unspecialized) and the *uenoi* species group (male fourth abdominal terga specialized).

##### Differential diagnosis.

Gravely (1910) compared the wing veins of *Alluaudellinahimalayensis* and *Cardacuswilleyi*, and concluded that the wing veins cannot be an important feature to distinguish these two genera. He distinguished the two genera by the presence or absence of ocelli, and whether the head is exposed or covered by the pronotum. Karny (1924) distinguished *Cardacopsis*, *Alluaudellina* and *Cardacus* by the degree of eye development, wing venation, and the presence or absence of ocelli, but he did not include *Nocticola* in the discussion. Roth (1988) considered that Chopard (1946) established the genus *Typhloblattodes* using a nymph specimen, questioning the validity of this genus. Chopard (1932, 1946, 1966) believed that there was polymorphism in the wing veins of *Alluaudellinahimalayensis*, and, in *Nocticola*, polymorphism in wings and eyes development, making these features of questionable value in generic diagnosis. Consequently, he was unable to distinguish *Nocticola* from *Alluaudellina* and believed that they may be synonyms. The genus *Nocticola* can be distinguished from *Typhloblatta* and *Pholeosilpha* by the following characteristics: anteroventral margin of front femur Type C, in contrast forefemoral spination type B2 in *Typhloblatta* and *Pholeosilpha*. The genus *Nocticola* can be distinguished from *Spelaeoblatta* by the following characteristics: 1) anteroventral margin of front femur Type C, in contrast fore femoral spination type B1 in *Spelaeoblatta*; 2) abdominal terga are unspecialized or have a gland on the fourth segment, while in *Spelaeoblatta* tergal glands on the second and third abdominal tergum; 3) female apterous, whereas female of *Spelaeoblatta* lack hind wings but have reduced lateral tegminal pads; 4) male tegmina membranous, with distinctive venation, while in *Spelaeoblatta* tegmina corneous, with poorly defined veins. The genus *Nocticola* can be distinguished from *Helmablatta* by the following characteristics: 1) anteroventral margin of front femur Type C, in contrast fore femoral spination type intermediate between A1 and B1 in *Helmablatta*; 2) abdominal terga are unspecialized or have a gland on the fourth segment, while in *Helmablatta* third to fifth abdominal tergum form a composite gland, and eighth abdominal tergum is specialized. The genus *Nocticola* can be distinguished from *Metanocticola* by the following characteristics: abdominal terga are unspecialized or have a gland on the fourth segment, while in *Metanocticola* male has a sex gland on the metanotum. According to current molecular analysis research, it has been shown that *Nocticola* is polyphyletic (Kovacs et al. 2024). The three new species are placed in the genus *Nocticola* based on features of the eyes, male wings, anteroventral margin of front femur type, arolia, pulvilli, abdomen tergal, and male genitalia.

### ﻿Key to species of *Nocticola* from China (males)

**Table:** 

1	Fourth abdominal tergum specialized, with dense setae on median area	***N.appendiculata* sp. nov.**
–	Fourth abdominal tergum not specialized	**2**
2	Tegmina not extending beyond the end of abdomen	***N.sinensis* Silvestri, 1946**
–	Tegmina extending beyond the end of abdomen	**3**
3	Tegmina and wings almost equal in length	**4**
–	Tegmina developed; hind wings reduced	***N.baiguensis* sp. nov.**
4	Subgenital plate weakly asymmetrical; accessory hook-like phallomere (L4N) parabola-like	***N.xiai* Liu, Zhu, Dai & Wang, 2017**
–	Subgenital plate symmetrical; accessory hook-like phallomere (L4N) fin-shaped	***N.cordiformis* sp. nov.**

#### ﻿ Nocticola sinensis

Taxon classificationAnimaliaBlattodeaNocticolidae

Silvestri, 1946

7E1E7D49-BFD5-55CE-8C30-6F1501954424

Fig. 2A–L

Nocticola sinensis Silvestri, 1946: 329; Silvestri 1947: 15; Princis 1952: 43; Princis 1966: 603; Roth 1988: 299; Liu et al. 2017.

##### Material examined.

China • 1 ♂ (deposited in SHEM); Hong Kong • 1 ♂, (deposited in ZSCTC), Hunan Province, Yongzhou City, Jiangyong County; 25°20.51'N, 111°20.34'E; 330 m; 14 July 2024, Hao-fei Fan leg.

##### Description.

Small size, adult yellowish. Male. (Fig. 2A, B). ***Head***: vertex of head exposed (Fig. 2C); eyes reduced to a few ommatidia narrowly grouped near antennal sockets (Fig. 2D); ocelli absent. Pronotum suboval, middle of posterior margin with weak invagination, anterior margin, and lateral margin with 12 setae (Fig. 2C). ***Tegmina and hind wings***: tegmina not extending beyond the end of abdomen (Fig. 2A), veins reduced in number, densely pubescent (Fig. 2F). Hind wings reduced, veins reduced in number (Fig. 2G). ***Legs***: legs long and slender. Anteroventral margin of front femur Type C1 (Fig. 2E); the first tarsus of the hind leg longer than the sum of the remaining tarsi; tarsal claws symmetrical and unspecialized (Fig. 2H), arolium and pulvillus absent (Fig. 2I). ***Abdomen and genitalia***: abdominal tergal gland unspecialized. Supra-anal plate symmetrical, middle of the hind margin triangular concave. Cerci 10 segments; ventral surface of segments not spinous setae (Fig. 2J). Subgenital symmetrical, middle of the hind margin concave (Fig. 2L). Style absent. Male genitalia: genital hook (L3) elongate, ventral to hook with approximately 14 strong setae; accessory hook-like phallomere (L4N), curved inward to approach a right angle, heavily sclerotized; L2 narrow; left process of L1 extends downward, and the right process curved inward; R1 membranous; R2 surface with scale-like tubercles; R3 membranous; R1H reduced, rounded margin (Fig. 2K).

##### Measurements (mm).

Male, pronotum: length × width: 0.76–0.90 × 0.98–1.20, tegmen: 1.73–2.00, body length (the length from the tip of vertex up to the tip of abdomen): 2.70–3.00;

##### Distribution.

China (Hong Kong, Hunan Prov.).

#### ﻿ Nocticola baiguensis sp. nov.

Taxon classificationAnimaliaBlattodeaNocticolidae

CA279ABF-1D85-553A-8D9A-532731C3FF62

https://zoobank.org/63BC7718-864D-4327-84F6-9433E063A45D

Figs 3A–M , 4A–F

##### Type material.

***Holotype***: China • 1 ♂; Guangxi Province, Guilin City, Yanshan District, Wangjia Village, Baigu Cave; 25°13.85'N, 110°20.52'E; 162 m; 1 November 2023, Hao-fei Fan leg; ZSCTC-LI0001. ***Paratype***: China • 9 ♂; same data as for holotype; 14 March 2024, Ting-Ting Li leg; ZSCTC-LI-0002- LI-00010 • 8 ♀; same collection data as for preceding; 14 March 2024, De-Xing Liu leg; ZSCTC-LI-00011- LI-00018 • 6 nymphs; same collection data as for preceding; 14 March 2024, Ting-Ting Li leg; ZSCTC-LI-00019- LI-00024 • 1 ♂; Guangxi Province, Guilin City, Xiangshan District, Guilin National Forest Park in Guanxi; 25°13.93'N, 110°13.92'E; 148 m; 18 March 2024, Hao-fei Fan leg; ZSCTC-LI-0025 • 1 ♂; Guangxi Province, Guilin City, Diecai District, Baiyun Temple, 25°18.75'N, 110°22.38'E; 526 m; 28 March 2024, Hao-fei Fan leg; ZSCTC-LI-0026 • 1 ♀; same collection data as for preceding; 28 March 2024, Hao-fei Fan leg; ZSCTC-LI-0027

##### Diagnosis.

**Figure 3.**
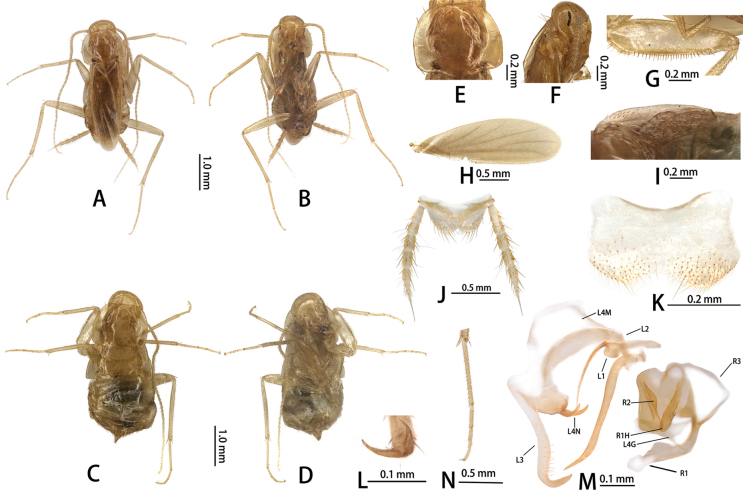
*Nocticolabaiguensis* sp. nov.: adult male **A** dorsal view **B** ventral view; adult female **C** dorsal view **D** ventral view **E** male pronotum **F** male head **G** forefemur **H** tegmen **I** hindwings **J** supra-anal plate, ventral view **K** subgenital plate, ventral view **L** tarsal claw **N** hind tarsus; male genitalia **M** phallomeres. Abbreviations: L1, L2, L3, L4G, L4N, L4M: sclerites of the left phallomere (L1 situated in the central dorsal wall; L2 arch-shaped sclerite situated in the ventral to L1; L3 situated in the left wall protrudes a large hook-process; L4G situated in the posterior ventral wall of the ventral lobe; L4N accessory hook-like phallomere; L4M situated in the ventral wall); R1, R2, R3, R1H: sclerites of the left phallomere (R1 situated in the dorsal or ventral walls, or in the posterior part of dorsal and ventral wall; R2 a ridge on the ventral margin; R3 plate-like situated in the anteriormost ventral wall; R1H a larger lobed situated in the dorsal wall, with extensions into the ventral wall).

The absence of a specialized abdominal tergal gland places the new species into the *simoni* species group. This species is similar to *N.baumi* Lucañas, Blaha, Rahmadi & Patoka, 2021, *N.bolivari* Chopard, 1950, *N.brooksi* Roth, 1995, *N.cockingi* Trotter, McRae, Main & Finston, 2017, *N.gonzalezi* Lucañas & Lit, 2016, *N.leleupi* Chopard, 1966, *N.quartermaieni* Trotter, McRae, Main & Finston, 2017, and *N.termitophila* Silvestri, 1946 as all these species are apterous. It can be distinguished by its eyes reduced to a few ommatidia narrowly grouped near antennal sockets (Fig. 3D), while in *N.baumi*, *N.bolivari*, and *N.cockingi* eyes are absent. In addition, the tegmina of this species are distinctly longer than the end of the abdomen, while in *N.brooksi*, *N.gonzalezi*, *N.leleupi*, *N.quartermaieni*, and *N.termitophila* tegmina do not extend beyond the end of abdomen.

##### Measurements (mm).

**Male**, pronotum: length × width: 0.80–1.01 × 1.09–1.30, tegmen: 2.29–2.32, overall length (including tegmen): 2.99–3.02, body length (the length from the tip of vertex up to the tip of abdomen): 2.85–3.35. Female, pronotum: length × width: 0.96–1.15 × 1.30–1.51, body length (the length from the tip of vertex up to the tip of abdomen): 3.16–3.67.

##### Description.

**Figure 4.**
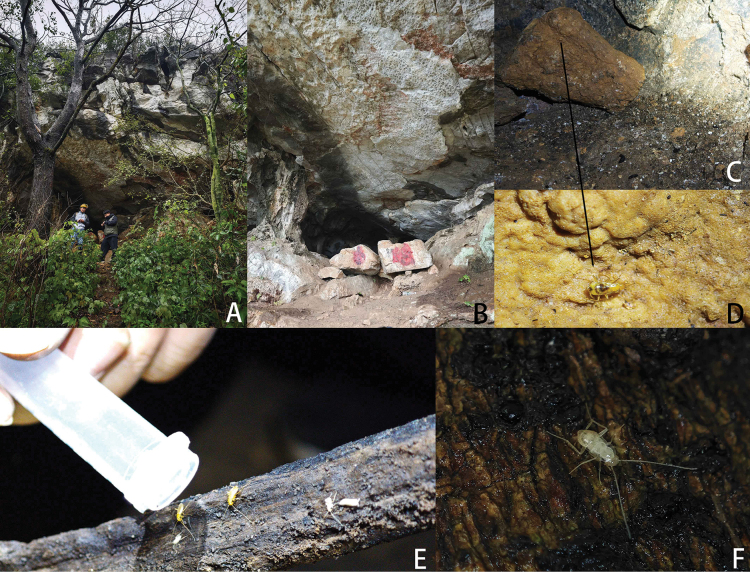
Habitats of *Nocticolabaiguensis* sp. nov. from China **A, B** external environment of the Baigu Cave **C, D***N.baiguensis* sp. nov. found on stone, surrounded by some animal feces **E***N.baiguensis* sp. nov. found on rotting branch **F** nymphs of *Nocticolabaiguensis* sp. nov.

Small size. Nymphs whitish (Fig. 4F), adult yellowish. Male. (Figs 3A, B, 4E). ***Head***: vertex of head exposed; eyes reduced to a few ommatidia narrowly grouped near antennal sockets (Fig. 3F); ocelli absent (Fig. 3F). Pronotum suboval, both sides of posterior margin with weak invagination, anterior margin and lateral margin with 12 setae (Fig. 3E). ***Tegmina and hind wings***: tegmina extending beyond the end of abdomen (Fig. 3A, B), veins reduced in number, densely pubescent (Fig. 3H). Lateral portions of metanotum pubescent, somewhat produced, suggesting a wing surface, but this region not separated from metanotum. ***Legs***: legs long and slender (Fig. 3I). Anteroventral margin of front femur Type C1 (Fig. 3G); the first tarsus of the hind leg longer than the sum of the remaining tarsi; tarsal claws symmetrical and unspecialized (Fig. 3L), arolium and pulvillus absent (Fig. 3N). ***Abdomen and genitalia***: abdominal tergal gland unspecialized. Supra-anal plate symmetrical, middle of the hind margin triangular concave. Cerci with 11 segments; ventral surface of segments without spinous setae (Fig. 3J). Subgenital symmetrical, middle of the hind margin concave (Fig. 3K). Style absent. Male genitalia: genital hook (L3) elongate, ventral to hook with approximately 17 strong setae; accessory hook-like phallomere (L4N), apex short and with double-hook, heavily sclerotized; L2 narrow, L1 elongate, process long spine-like; R1 membranous, distal capitate-like; R2 sinuate protrusion of central part, surface with scale-like tubercles; R3 membranous; R1H reduced, rounded margin (Fig. 3M).

**Female**: Apterous (Fig. 4C, D). Supra-anal plate triangular, transverse of hind margin, middle with U-shaped invagination. Subgenital lobate (Fig. 3C, D). Cerci with 11 segments; ventral surface of segments without spinous setae.

##### Etymology.

The specific name *baiguensis* is derived from the cave called Baigu Cave (Fig. 4A, B), which is the first collection site of this species.

##### Distribution.

China (Guangxi).

#### ﻿ Nocticola cordiformis sp. nov.

Taxon classificationAnimaliaBlattodeaNocticolidae

FCBA08CF-1C1B-59E3-B5E2-A8D5381EC11A

https://zoobank.org/867C3F19-3F52-4B41-80D9-8ECE341029E7

Figs 5A–M , 6A–C

##### Type material.

***Holotype***: China • 1 ♂; Guangxi Province, Guilin City, Lingui District, Huixian Town, Edi Village, Yanzi Cave; 25°3.30'N, 110°11.27'E; 195 m; 13 December 2023; Hao-Fei Fan leg; ZSCTC-LI-0028**. *Paratype***: China • 1 ♂; Guangxi Province, Guilin City, Lingui District, Lingjiaodi Village; 25°13.58'N, 110°14.50'E; 148 m; 30 December 2023; Hao-Fei Fan leg; ZSCTC-LI-0029 • 1 ♀; same collection data as for preceding; 30 December 2023; Hao-Fei Fan leg; ZSCTC-LI-0030 • 2 ♂; Guangxi Province, Guilin City, Lingui District, Huixian Town, Edi Village, 25°3.07'N, 110°10.25'E; 176 m; 29 April 2024; Hao-Fei Fan leg; ZSCTC-LI-0031 to 0032.

##### Diagnosis.

**Figure 5.**
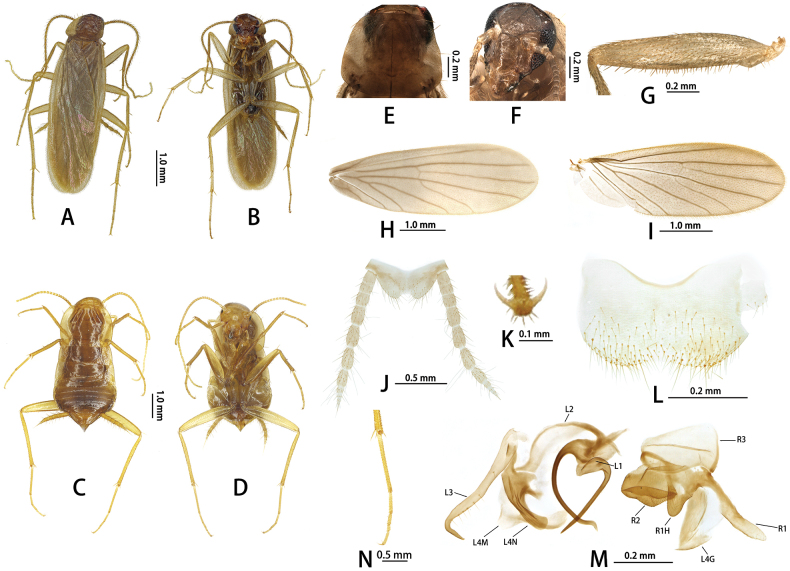
*Nocticolacordiformis* sp. nov.: **A** male, dorsal view **B** male, ventral view **C** female, dorsal view **D** female, ventral view **E** pronotum **F** head **G** front femur **H** tegmen **I** wings **J** supra-anal plate, ventral view **K** tarsal claw **L** subgenital plate, ventral view **N** hind tarsus; male genitalia, **M** phallomeres. Abbreviations: L1, L2, L3, L4G, L4N, L4M sclerites of the left phallomere (L1 situated in the central dorsal wall; L2 arch-shaped sclerite situated in the ventral to L1; L3 situated in the left wall protrudes a large hook-process; L4G situated in the posterior ventral wall of the ventral lobe; L4N accessory hook-like phallomere; L4M situated in the ventral wall); R1, R2, R3, R1H sclerites of the left phallomere (R1 situated in the dorsal or ventral walls, or in the posterior part of dorsal and ventral wall; R2 a ridge on the ventral margin; R3 plate-like situated in the anteriormost ventral wall; R1H a larger lobed situated in the dorsal wall, with extensions into the ventral wall).

The absence of a specialized abdominal tergal gland places the new species into the *simoni*-species group. This species is similar to *N.adebratti* Roth, 1994, *N.babindaensis* Roth, 1994, *N.clavate* Andersen & Kjaerandsen, 1995, *N.gerlachi* Roth, 2003, *N.pheromosa* Lucañas & Maosheng, 2023, *N.scytale* Andersen & Kjaerandsen, 1995, *N.wliensis* Andersen & Kjaerandsen, 1995, and *N.xiai* Liu, Zhu, Dai & Wang, 2017, with tegmina and wings developed. It differs from *N.adebratti* and *N.gerlachi* by its ocelli absent (Fig. 5F), while in *N.adebratti* and *N.gerlachi* ocelli are present. The subgenital plate of the newly described species is symmetrical (Fig. 5L), while in *N.adebratti*, *N.babindaensis*, *N.clavate*, *N.gerlachi*, *N.pheromosa*, *N.scytale*, and *N.wliensis*, the subgenital plate is asymmetrical. It differs from *N.xiai* as follows: 1) tegmina and wings extending beyond the end of abdomen, body length is about half of the wing length, while in *N.xiai* tegmina and wings slightly extend beyond the end of abdomen, but not exceeding half of the body length (Fig. 1A); 2) eyes well developed, while in *N.xiai* eyes reduced (Fig. 1B); and 3) accessory hook-like phallomere (L4N) fin-shaped, whereas L4N is parabola-like in *N.xiai* (Fig. 1C).

##### Measurements (mm).

Male, pronotum: length × width: 0.81–1.02 × 0.99–1.21, tegmen: 4.04–4.25, overall length (including tegmen): 4.83–5.24, body length (the length from the tip of vertex up to the tip of abdomen): 3.19–3.27. Female, pronotum: length × width: 1.13–1.22 × 1.35–1.42, body length (the length from the tip of vertex up to the tip of abdomen): 3.45–3.66.

##### Description.

**Figure 6.**
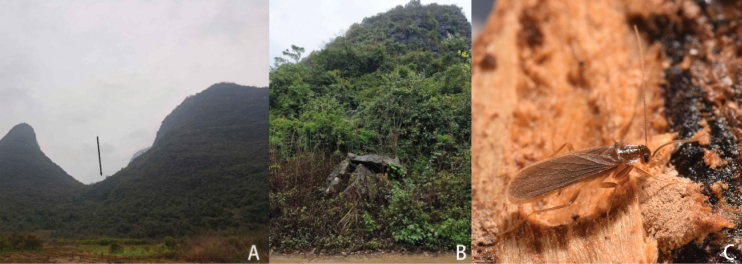
Habitats of *Nocticolacordiformis* sp. nov. from China **A, B** habitats of holotype specimen of *N.cordiformis* sp. nov. **C** adult of *N.cordiformis* sp. nov. on wood.

Small size. Body tawny. **Male.** (Figs 5A, B, 6C). ***Head***: vertex of head exposed; Eyes well developed, with heptagon concave; ocelli absent (Fig. 5F). Pronotum subtrapezoidal, densely pubescent, anterior margin and lateral margin with 12 setae (Fig. 5E). ***Tegmina and hind wings***: tegmina and wings well developed, extending beyond the end of abdomen, body length is about half of the wing length (Fig. 5A, B), veins reduced in number, densely pubescent (Fig. 5H, I). ***Legs***: legs long and slender. Anteroventral margin of front femur Type C1 (Fig. 5G); the first tarsus of the hind leg longer than the sum of the remaining tarsi; tarsal claws symmetrical and unspecialized (Fig. 5K), arolium and pulvillus absent (Fig. 5N). ***Abdomen and genitalia***: abdominal tergal gland unspecialized. Supra-anal plate symmetrical, middle of the hind margin concave (Fig. 5J). Subgenital plate symmetrical, middle of the hind margin weakly concave (Fig. 5L). Style absent. Male genitalia: L3 elongate, ventral to hook with 7 strong setae; L4N fin-shaped; L2 narrow; left and right process of L1 towards curved, heart-shape; R1 long handle-like, covered with some setae; R2 sinuate protrusion, with scale-like tubercles; R3 membranous; R1H reduced, rounded margin, with scale-like tubercles (Fig. 5M).

**Female**: Apterous. Supra-anal plate triangular, transverse of hind margin, middle with triangular invagination. Subgenital lobate (Fig. 5C, D). Cerci 11 segments; ventral surface of segments not spinous setae.

##### Etymology.

The specific name is derived from the Latin word *cordiformis*, in reference to the L1 heart-shaped structure of left aedeagus.

##### Distribution.

China (Guangxi).

#### ﻿ Nocticola appendiculata sp. nov.

Taxon classificationAnimaliaBlattodeaNocticolidae

FEB017E3-866B-546B-AD90-DB3D801BE14C

https://zoobank.org/C49BF683-1E94-4EFF-8E5A-E49684049B8C

Figs 7A–P , 8A–D

##### Type material.

***Holotype***: China • 1 ♂; Guangxi Province, Guilin City, Lingchuan County, Haiyang Town, Xiaofu Village, 25°15.17'N, 110°35.33'E; 329 m; 26 April 2024; Hao-Fei Fan leg; ZSCTC-LI-0033. ***Paratype***: China • 1 ♂, 1 ♀; same data as for holotype; Hao-Fei Fan leg; ZSCTC-LI-0034 to 0035 • 2 ♂; Guangxi Province, Guilin City, Lingui District, Huixian Town, Edi Village, 25°3.67'N, 110°10.25'E; 176 m; 29 April 2024; Hao-Fei Fan leg; ZSCTC-LI-0036 to 0037.

##### Diagnosis.

The fourth abdominal tergum specialized tergal gland places the new species into the *uenoi*-species group. It closely resembles *Nocticolacurrani* Trotter, McRae, Main & Finston, 2017 in terms of the well-developed tegmina and fourth abdominal tergum specialized. It differs from *N.currani* as follows: 1) wings curved, longer than tegmina and extending beyond the end of abdomen, while in *N.currani* wings reduced, not extending beyond the first abdomen; 2) ventral of L3 hook with ~ 10 strong setae scattered, while in *N.currani* with 11 long and strong setae clustered on distal end below the curve of L3 hook; and 3) accessory hook-like phallomere (L4N) inner margin curved and smooth at distal end, whereas L4N is spear-shaped, with longitudinal ribbing of distal end in *N.currani*. In addition, the tegmina of this species are distinctly longer than the end of the abdomen, with a small appendicular field, while the tegmina of *N.australiensis* Roth, 1988, *N.uenoikikaiensis* Asahina, 1974, *N.uenoimiyakoensis* Asahina, 1974, *N.uenoiuenoi* Asahina, 1974 and *N.rohini* (Fernando, 1962) are shorter than the end of the abdomen, and without appendicular field.

##### Measurements (mm).

Male, pronotum: length × width: 1.01–1.21 × 1.08–1.29, tegmen: 2.66–2.87, wings: 3.05–3.26, overall length (including tegmen): 4.08, body length (the length from the tip of vertex up to the tip of abdomen): 2.48–2.65. Female, pronotum: length × width: 1.10 × 1.33; body length (the length from the tip of vertex up to the tip of abdomen): 3.50.

##### Description.

**Figure 7.**
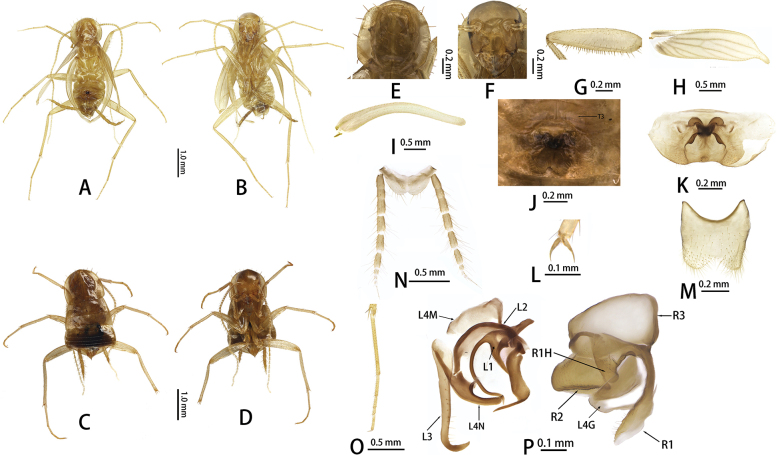
*Nocticolaappendiculata* sp. nov. **A** male, dorsal view **B** male, ventral view **C** female, dorsal view **D** female, ventral view **E** pronotum **F** head **G** front femur **H** tegmen **I** wings **J, K** male T4, dorsal view **L** tarsal claw **N** supra-anal plate, ventral view **M** subgenital plate, dorsal view **O** hind tarsus, male genitalia **P** phallomeres. Abbreviations: L1, L2, L3, L4G, L4N, L4M: sclerites of the left phallomere (L1 situated in the central dorsal wall; L2 arch-shaped sclerite situated in the ventral to L1; L3 situated in the left wall protrudes a large hook-process; L4G situated in the posterior ventral wall of the ventral lobe; L4N accessory hook-like phallomere; L4M situated in the ventral wall); R1, R2, R3, R1H: sclerites of the left phallomere (R1 situated in the dorsal or ventral walls, or in the posterior part of dorsal and ventral wall; R2 a ridge on the ventral margin; R3 plate-like situated in the anteriormost ventral wall; R1H a larger lobed situated in the dorsal wall, with extensions into the ventral wall).

**Figure 8.**
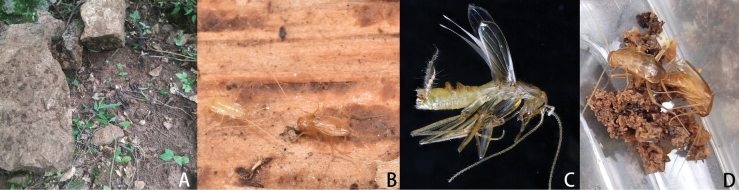
Habitats of *Nocticolaappendiculata* sp. nov. from China **A** habitats of *N.appendiculata* sp. nov. **B** nymphs and an adult of *N.appendiculata* sp. nov. on wood **C***N.appendiculata* sp. nov., side view **D** female and male of *N.appendiculata* sp. nov. mating.

Small size. Nymphs whitish (Fig. 8B). Body yellowish. **Male** (Figs 7A, B, 8B, C, D). ***Head***: vertex of head exposed; eyes reduced; ocelli absent (Fig. 7D). Pronotum ovoid, densely pubescent, hind margin weakly concave, anterior and lateral margins with 12 setae (Fig. 7B). ***Tegmina and hind wings***: tegmina extending beyond the end of abdomen, with a small appendicular at the apical (Fig. 7H). Wings inflexion, longer than tegmina and extending beyond the end of abdomen, veins indistinct, with some setae. Hind wings are placed on both sides of the abdomen and not covered by the tegmina (Fig. 7A, B, I). **Legs**: legs long and slender. Anteroventral margin of front femur Type C1 (Fig. 7G); the first tarsus of the hind leg longer than the sum of the remaining tarsi; tarsal claws symmetrical and unspecialized (Fig. 7L), arolium and pulvillus absent (Fig. 7O). ***Abdomen and genitalia***: second and third abdominal tergum deeply concave on hind margin, exposing the gland of the fourth abdominal tergum. Fourth abdominal tergum specialized, median area has dense setae convex and a large and deep invagination; Posterior margin has three convexities, with the middle one being smaller than the two sides (Fig. 7J, K). Supra-anal plate symmetrical, middle of the hind margin concave (Fig. 7N). Subgenital plate symmetrical, middle of the hind margin weakly concave (Fig. 7M). Style absent. Male genitalia of left phallomere: L3 elongate, ventral to hook with ~ 10 strong setae scattered; L4N inner margin smooth; L2 narrow; L1 long, apex of left process slightly curved towards L4N. Male genitalia of right phallomere: R1 medial border with several long setae; R2 sinuate protrusion, with scale-like tubercles; R3 membranous; R1H reduced, rounded margin, with scale-like tubercles (Fig. 7P).

**Female**: Apterous (Fig. 8D). Supra-anal plate triangular, transverse of hind margin, U-shaped invagination slightly to the right in the middle of hind margin. Subgenital lobate (Fig. 7C, D). Cerci with 11 segments; ventral surface of segments without spinous setae.

##### Etymology.

The specific name is derived from the Latin word *appendiculatus*, in reference to the extended appendicular field of tegmina.

##### Distribution.

China (Guangxi).

### ﻿Molecular analysis based on COI

We collected the 14 COI sequences of the three new species and blasted them on GenBank. The resulting sequence alignment comprised 658 nucleotides. Sequence comparisons reveal sequence differences in nucleotide numbers (Table 2, above diagonal), as well as intra- and interspecific genetic distances (Table 2, below diagonal). The intraspecific genetic distance of these three new species is 0. The interspecific genetic distance between *N.baiguensis* sp. nov. and *N.cordiformis* sp. nov. is 19.40%, while that between *N.baiguensis* sp. nov. and *N.appendiculata* sp. nov. is 21.05%; The genetic distance between *N.appendiculata* sp. nov. and *N.cordiformis* sp. nov. is 4.26%. The COI sequence of these two species differ in 27 nucleotide sites.

**Table 2.** Genetic distances (below diagonal) and nucleotide sites differences (above diagonal) of three new species based on COI sequences.

Specimen	LI49-1	LI49-2	LI69-1	LI69-2	LI70	LI70-1	LI70-2	LI51	LI85	LI86	LI95	LI96	LI81	LI82
LI49-1*N.baiguensis* sp. nov.		0	0	0	0	0	0	112	112	112	112	112	120	120
LI49-2 *N.baiguensis* sp. nov.	0.0000		0	0	0	0	0	112	112	112	112	112	120	120
LI69-1 *N.baiguensis* sp. nov.	0.0000	0.0000		0	0	0	0	112	112	112	112	112	120	120
LI69-2 *N.baiguensis* sp. nov.	0.0000	0.0000	0.0000		0	0	0	112	112	112	112	112	120	120
LI70 *N.baiguensis* sp. nov.	0.0000	0.0000	0.0000	0.0000		0	0	112	112	112	112	112	120	120
LI70-1 *N.baiguensis* sp. nov.	0.0000	0.0000	0.0000	0.0000	0.0000		0	112	112	112	112	112	120	120
LI70-2 *N.baiguensis* sp. nov.	0.0000	0.0000	0.0000	0.0000	0.0000	0.0000		112	112	112	112	112	120	120
LI51 *N.cordiformis* sp. nov.	0.1940	0.1940	0.1940	0.1940	0.1940	0.1940	0.1940		0	0	0	0	27	27
LI85 *N.cordiformis* sp. nov.	0.1940	0.1940	0.1940	0.1940	0.1940	0.1940	0.1940	0.0000		0	0	0	27	27
LI86 *N.cordiformis* sp. nov.	0.1940	0.1940	0.1940	0.1940	0.1940	0.1940	0.1940	0.0000	0.0000		0	0	27	27
LI95 *N.cordiformis* sp. nov.	0.1940	0.1940	0.1940	0.1940	0.1940	0.1940	0.1940	0.0000	0.0000	0.0000		0	27	27
LI96 *N.cordiformis* sp. nov.	0.1940	0.1940	0.1940	0.1940	0.1940	0.1940	0.1940	0.0000	0.0000	0.0000	0.0000		27	27
LI81 *N.appendiculata* sp. nov.	0.2105	0.2105	0.2105	0.2105	0.2105	0.2105	0.2105	0.0426	0.0426	0.0426	0.0426	0.0426		0
LI82 *N.appendiculata* sp. nov.	0.2105	0.2105	0.2105	0.2105	0.2105	0.2105	0.2105	0.0426	0.0426	0.0426	0.0426	0.0426	0.0000	

**Figure 9.**
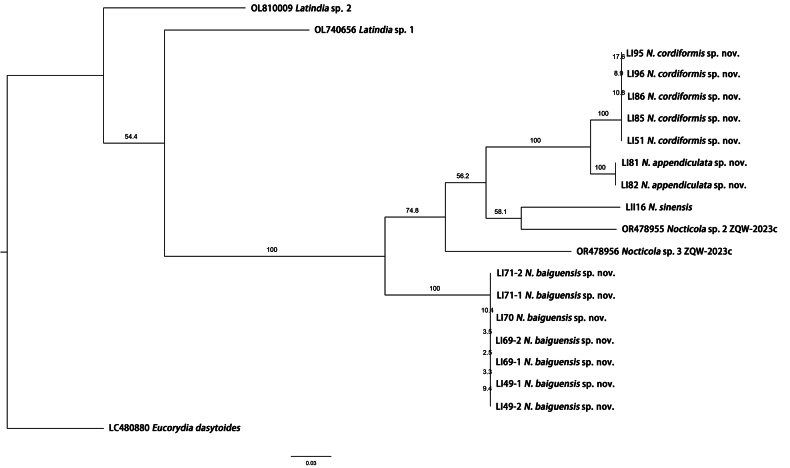
Maximum likelihood (ML) tree of *N.baiguensis* sp. nov., *N.cordiformis* sp. nov. and *N.appendiculata* sp. nov. based on a portion of the COI sequence. Bootstrap values are reported above each branch.

The phylogenetic tree of the 20 COI sequences, derived from Table 1, is depicted in Fig. 9. The maximum likelihood (ML) tree shows that samples with the same morphology exhibit high bootstrap values in forming monophyletic groups, although most of the other nodes did not have high bootstrap values.

## ﻿Discussion

In this study, we used morphological differential diagnosis to conclude that *N.baiguensis*, *N.cordiformis*, and *N.appendiculata* are new species of the genus *Nocticola*. The three species have significant differences in male genitalia. Molecular comparison of these three species show that the maximum genetic distance between them was 21.05% (*N.baiguensis* and *N.cordiformis*), while the minimum interspecies genetic distance was 4.26% (*N.cordiformis*, and *N.appendiculata*).

*Nocticola* is mainly distributed in Africa, Asia, and Australasia (Beccaloni 2014), with two species *N.xiai* Liu, Zhu, Dai & Wang, 2017 and *N.sinensis* Silvestri, 1946 recorded in China, both of which are termitophilous (Silvestri 1946; Liu et al. 2017); the other two termitophilous species are *N.termitophila* Silvestri, 1946 and *N.jodarlingtonae* Roth, 2003 (Silvestri 1946; Roth 2003a). The four species of *Nocticola* were found in termite nests, but at that time, there were no research records of a relationship between *Nocticola* and termites. In March 2023, we discovered a female *Nocticola* sp. with reduced eyes in Jiangjunshan Park, Zhuhai City, Guangdong Province. It was found in a relatively dry, mud-built, ant nest at the bottom of some decaying wood; the ant species was identified as *Camponotusnicobarensis* Mayr, 1865. Unfortunately, we only found one female *Nocticola* sp. Due to the scarcity of evidence, we cannot determine whether this *Nocticola* sp. accidentally entered the nest or had a relationship with *C.nicobarensis*.

This article describes three new species: *N.baiguensis* sp. nov., *N.cordiformis* sp. nov., and *N.appendiculata* sp. nov. Among them, *N.baiguensis* sp. nov. was first discovered in the Baigu Cave, which is a natural karst cave. We found them on some decaying branches and stones next to the bat feces. We collected four pairs of *N.baiguensis* sp. nov. in the cave, and one of them was surrounded by many small nymphs. We brought them back to the laboratory for breeding. Kept under observation, we found that they feed on mycelium on wood at a slow rate. They prefer to hide under the bottom of wood and wet tissues and did not seem to be very active. We also found some ants identified as *C.nicobarensis* living together with *N.baiguensis* sp. nov.

China is a country with the largest area of karst caves, a total of approximately 3.4 million square kilometers and approximately 500,000 karst caves (Zhang et al. 2021). Most of the karst caves have constant temperature and humidity inside because of the underground space with dark environments and complex terrain (Ran 2013). Due to some social and historical reasons, the exploration of cave-dwelling organisms started relatively late in China (Zhang 1992), resulting in few reports on cave insects and the present research on cave cockroaches. *Nocticolacordiformis* sp. nov. and *N.appendiculata* sp. nov. were found under rocks at the roadside. The eyes and wings of *N.cordiformis* sp. nov. are well developed, while the eyes of *N.baiguensis* sp. nov. and *N.appendiculata* sp. nov. were smaller. *Nocticolaappendiculata* sp. nov. has well-developed front and hind wings, whereas tegmina are well developed and hind wings reduced in *N.baiguensis* sp. nov. During the sample collection process, it was common to find *N.cordiformis* sp. nov. and *N.appendiculata* sp. nov. living under the same stone, with overlapping areas, although they have certain differences in morphology and COI sequences. Further research on the living habits of *Nocticola* and exploration of their relationship with ants should be carried out in the future.

## Supplementary Material

XML Treatment for Nocticola

XML Treatment for Nocticola sinensis

XML Treatment for Nocticola baiguensis

XML Treatment for Nocticola cordiformis

XML Treatment for Nocticola appendiculata

## ﻿Additional information

### Author ORCIDs

Ting-Ting Li https://orcid.org/0000-0001-5127-5344

De-Xing Liu https://orcid.org/0009-0009-9946-9864

Jian Chen https://orcid.org/0000-0002-2025-7079

Xiao-Ya Wei https://orcid.org/0009-0007-3297-8109

### Data availability

All of the data that support the findings of this study are available in the main text.
